# Supplementary material for: Differences in a Single Extracellular Residue Underlie Adhesive Functions of Two Zebrafish Aqp0s
Source: Cells. 2021 Aug 6;10(8):2005. doi: 10.3390/cells10082005 (PMC8392583; doi:10.3390/cells10082005)
Supplement: Supplementary file 1 [file cells-10-02005-s001.zip › cells-1290029-supplementary.pdf]

Supplementary material

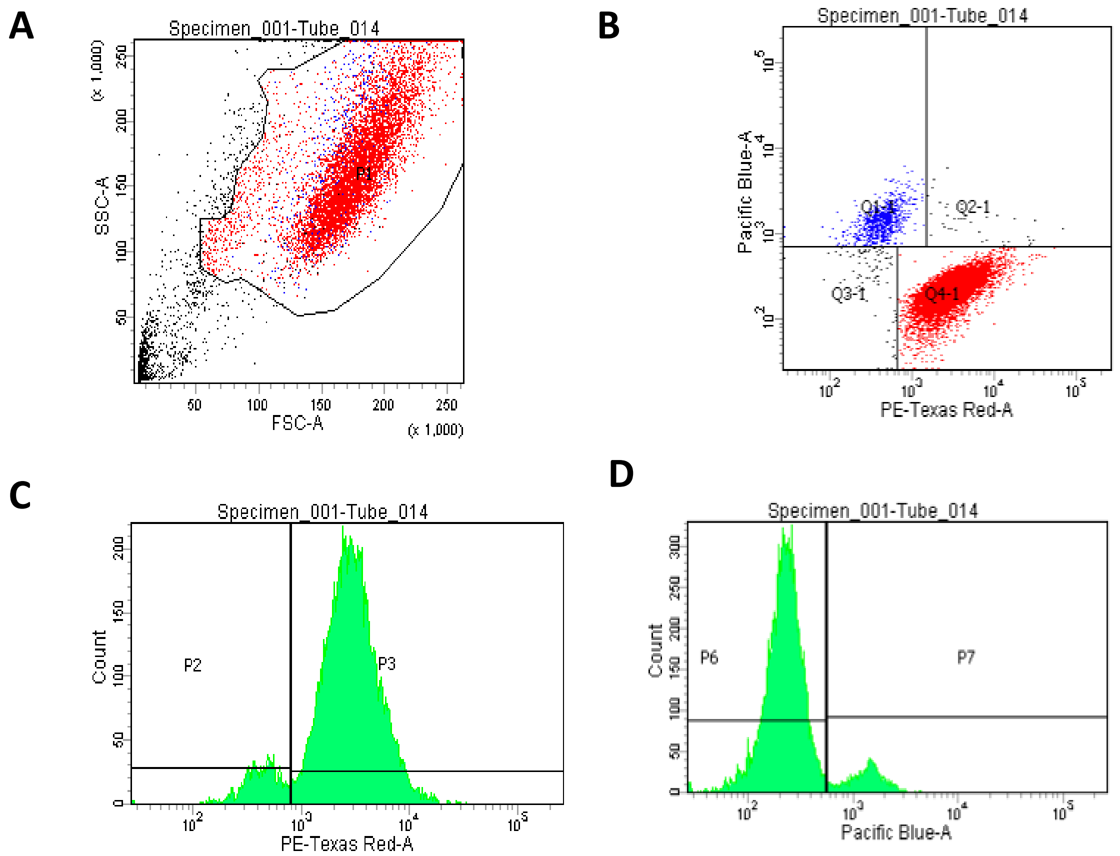

Supplementary Figure 1. Flow cytometry data example. Cell adhesion assay was done using flow cytometer. The L-cells expressing either Aqp0a or Aqp0b loaded with CellTracker Red dye were cultured as monolayer. After 18 hours, other cells loaded with CellTracker Blue seeded over the first monolayer. After washing off non-adherent cells, the intensity of fluorescence of CellTracker Red was detected as Texas Red filter and that of CellTracker Blue was detected as pacific blue filter. (A) Gating of measured cells. (B) Example of negative control rAQP1- rAQP1 reveals 10.3%. blue positive cells revealing poor adhesive properties. (C, D) Signal intensity of Texas Red (C) and Pacific Blue (D), respectively.

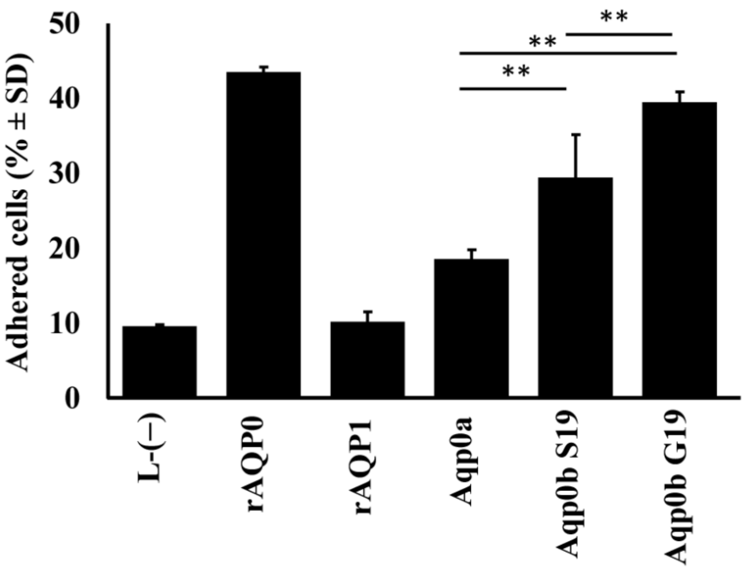

Supplementary Figure 2. Aqp0b S19 has auto-adhesive properties. The non-water permeable Aqp0b S19 variant has lower adhesive properties compared to Aqp0b G19, but higher than Aqp0a. Each lane represents n=6-8. Statistical differences are shown ** having $p < 0.01$.

Supplementary Table 1. Protein blast results of the anti-human AQP0 peptide sequence in *Danio rerio* and *Rattus norvegicus*. Santa Cruz H-44 antibody is directed against amino acids 220-263 (DFLLFPRLKSISERLSVLKGAKPDVSNGQPEVTGEPVELNTQAL) within the C-terminal domain of human AQP0. It is predicted to identify both, zebrafish Aqp0a and Aqp0b at similar affinity.

**Description**	**Scientific Name**	**Max Score**	**Total Score**	**Query Cover**	**E value**	**Per. ident**	**Acc. Len**	**Accession**
major intrinsic protein of lens fiber [Rattus norvegicus]	Rattus norvegicus	77	77	100%	2.00E-20	84.09	61	BAM83579.1
lens fiber major intrinsic protein isoform X1 [Rattus norvegicus]	Rattus norvegicus	80.1	80.1	100%	3.00E-20	84.09	185	XP_038934393.1
lens fiber major intrinsic protein [Rattus norvegicus]	Rattus norvegicus	81.3	81.3	100%	4.00E-20	84.09	263	NP_001099189.1
RecName: Full=Lens fiber major intrinsic protein; AltName: Full=Aquaporin-0; AltName: Full=MIP26; Short=MP26 [Rattus norvegicus]	Rattus norvegicus	80.9	80.9	100%	5.00E-20	84.09	261	P09011.2
major intrinsic protein of lens fiber a [Danio rerio]	Danio rerio	62.4	62.4	100%	4.00E-13	61.36	263	NP_001003534.1
major intrinsic protein of lens fiber b [Danio rerio]	Danio rerio	58.9	58.9	100%	8.00E-12	54.55	263	NP_001018356.1
Mip2 protein [Danio rerio]	Danio rerio	58.9	58.9	100%	8.00E-12	54.55	263	AAH98535.1
Mip2 protein [Danio rerio]	Danio rerio	58.9	58.9	100%	8.00E-12	54.55	263	AAI55723.1
RecName: Full=Aquaporin-2; [Rattus norvegicus]	Rattus norvegicus	38.1	38.1	93%	2.00E-04	47.62	271	P34080.1
aquaporin-2 [Rattus norvegicus]	Rattus norvegicus	38.1	38.1	93%	2.00E-04	47.62	271	NP_037041.3
unknown [Rattus norvegicus]	Rattus norvegicus	37.7	37.7	93%	3.00E-04	47.62	312	AAA41478.1
aquaporin 1a (Colton blood group), tandem duplicate 2 isoform X2 [Danio rerio]	Danio rerio	33.5	33.5	59%	0.012	55.56	269	XP_009297361.1
aquaporin-1b [Danio rerio]	Danio rerio	33.1	33.1	59%	0.015	55.56	269	ACA29537.1
aquaporin 1a (Colton blood group), tandem duplicate 2 isoform X1 [Danio rerio]	Danio rerio	33.1	33.1	59%	0.015	55.56	269	XP_021336241.1
